# Epimerization of ergot alkaloids in feed

**DOI:** 10.1016/j.heliyon.2020.e04336

**Published:** 2020-06-30

**Authors:** Claude Schummer, Irène Zandonella, An van Nieuwenhuyse, Gilbert Moris

**Affiliations:** Laboratoire National de Santé, Service de Surveillance Alimentaire, 1, rue Louis Rech, 3555 Dudelange Luxembourg

**Keywords:** Food analysis, Food safety, Food technology, Ergot alkaloids, Epimerization, Feed processing, UPLC-MS/MS

## Abstract

Chronic intake of cereals contaminated with ergot alkaloids can cause ergotism and result in the loss of toes and fingers or even death. Today, due to common risk management practices, ergotism is rare as a human disease but remains a problem in livestock husbandry. Each alkaloid coexists under two forms (R and S), though only the R-form presents toxic effects. The epimerization occurs spontaneously but the mechanisms remain globally unknown. Therefore, different processing methods were evaluated for their respective influences on the epimerization. The results suggest that ergotamine and ergosine are very stable ergot alkaloids, and neither their concentrations, nor their respective R/S ratios, are significantly influenced by heating, protic solvents or UV light. In contrast, for ergocristine, ergokryptine, ergocornine and ergometrine, heating can decrease the concentrations of these alkaloids and heat, protic solvents and UV light influence the R/S ratio towards the S-form, though the respective influence on the epimerization of these compounds is variable. In addition, the total concentration of all ergot alkaloids is reduced through heating. However, all these effects are not strong enough to change the composition of ergot alkaloids in feed substantially and to transform toxic feed into non-toxic feed.

## 1 Introduction

Ergot alkaloids are the active substances produced by some species of *Claviceps purpurea*, a sac fungi growing in moderate climate zones on sweet grasses like rye, triticale, wheat or sorghum, and on wild herbs (Geiger and Miedaner, 2009). In autumn, the fungus sends out a dark spur, called the ergot sclerotia, that falls to the ground during harvest, overwinters, and sends out spores in spring. The spores enter the open florets of the plant during bloom and infect the ovaries of the plants, and thus the fungus grows on the cereal grains producing new mycelium and sclerotia. Although most sweet grasses can be infected by *Claviceps purpurea*, rye and triticale are most concerned because they are self-pollinators and thus their blossoms open more widely and can collect more spores (Lorenz, 1979; Krska et al., 2008; Di Mavungu et al., 2012; Merkel, 2013; EFSA, 2012, 2017).

Humans are exposed to ergot alkaloids via food intake. Chronic and large intakes of ergot alkaloids can cause ergotism, an illness causing strange hallucinations, the feeling of itchy and burning skin, gangrene through undersupply of blood and as a consequence theloss of toes and fingers. Although ergotism was a big problem in medieval times where it was called St. Anthony's fire or Holy Fire (Breitmaier, 2008), it plays nearly no role in human medicine today, but remains a problem in livestock husbandry, above all for ruminants (Bennet and Klich, 2003). Furthermore, recent studies show that infections of rye with *Claviceps purpurea* have been increasing over the last ten years, probably due to the increased use of hybrid seed, all-season cultivation of rye and insufficient ploughing (Amelung, 1999; Engelke, 2002).

**Figure 1:**
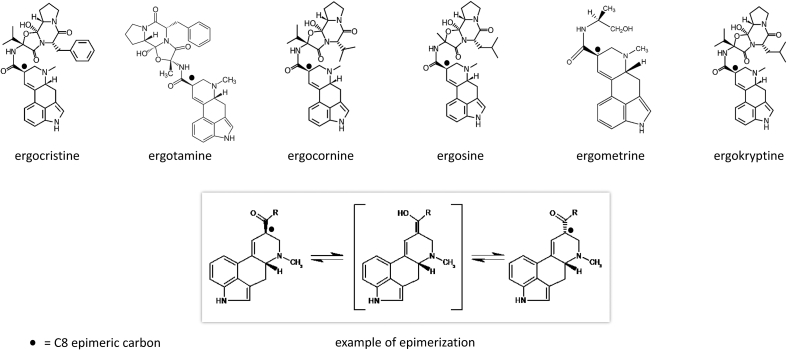
Structures of the studies ergot alkaloids (*-ine* form) with labelling of the C8-epimeric carbon, and schema of the keto-enol tautomerism (epimerization).

*Claviceps purpurea* produces more than 50 different alkaloids, of which ergotamine, ergocristine, ergosine, ergocornine, ergokryptine and ergometrine are the most important (EFSA, 2012). These alkaloids have a stereocenter on position C_8_ that exists in an R- or an S-configuration (Figure 1). In order to distinguish both epimers the R-form is characterized with a final syllable “ine” and the S-form with “inine” (e.g. ergotamine and ergotaminine). The ergopeptides can be converted via keto-enol tautomerism into ergopeptinines and vice versa (Figure 1), explaining why in nature both forms always coexist (Lehner et al., 2005). This fact is important because the R-epimer has a toxic effect on humans and mammals while the S-epimer has almost no pharmacological effect (Komarova and Tolkachev, 2001). The epimerization can be influenced by different parameters that must be considered during sample preparation. Thus, Krska et al. (2008) showed that extraction with acid or alkaline buffers favours epimerization, and Komarova and Tolkachev (2001) showed that above all alkaline buffers favour the epimerisation from R to S. Protic solvents, too, favour epimerization, only with chloroform no epimerization was observed (Hafner et al., 2008). It has also been observed in previous research projects that heating of the contaminated cereal products, like baking, favours epimerization and degradation of the ergot alkaloids (Mainka et al., 2005; Lampen and Klaffke, 2006; Lauber et al., 2005; Hafner et al., 2008). But globally, the knowledge about the epimerization mechanisms and the influences on it remain sparse, and it is yet not possible to control the epimerization. However, as the toxicity of both epimers is significantly different, exact knowledge of the R/S ratio in food and feed is important in order to perform accurate and precise risk assessment. Therefore, the aim of this study was to increase knowledge about the parameters influencing epimerization by investigating the influences of heat, humidity, pH, UV-light and matrix (different kinds of cereals and feed). Furthermore, if epimerization could be controlled, it might be possible to turn unhealthy, contaminated feed into unproblematic feed by converting all R-epimers into S-epimers.

After exploring the influences on epimerization, the findings were applied to naturally contaminated feed samples that were first analysed as-is and then re-analysed after processing of the samples in order to see if the R/S ratio and/or the concentrations had changed.

## 2 Materials and methods

### 2.1 Chemicals

Reference standards (ergotamine/ergotaminine, ergometrine/ergometrinine, ergosine/ergosinine, α-ergokryptine/α-ergokryptinine, ergocornine/ergocorninine, and ergocristine/ergocristinine) were purchased as dried down standards from Romer Labs (Tulln, Austria). Methanol, acetonitrile and acetone were purchased from Biosolve (Dieuze, France). Ultra-pure water was obtained using a Millipore lab water purification system (Overijse, Belgium). All other chemicals were purchased from VWR (Oud-Heverlee, Belgium).

Standard solutions of each compound were produced by adding 5 ml acetonitrile to 0.5 mg dried-down standard following the instructions of the producer, resulting in a concentration of 100 μg/ml. Working solutions containing all alkaloids were prepared weekly in acetonitrile. All solutions were stored at -20 °C as recommended by the producer, in order to prevent epimerization.

Preparation of acid buffer: 770.8 mg of ammonium acetate (purity >99%) were dissolved in 800 ml water, and pH was adjusted to 3 with acetic acid (>99 %). Afterwards the total volume was adjusted to 1 l with water.

Preparation of alkaline buffer: 6.18 g of boric acid and 4 g of potassium hydroxide were dissolved in 100 ml with water. The pH of this solution is about 10.

### 2.2 Samples

All samples analyzed in this study were sampled by the national agency for technical agricultural services (ASTA – Administration des services techniques de l′agriculture). The samples consisted of unprocessed grains of rye, triticale and wheat, sieved according to the standard ISO 5223 using a grain sieve with a 1.9 × 20.0 mm slotted stainless steel plate, and fodder pellets composed of different types of cereals and herbs.

### 2.3 Sample preparation

Samples received by ASTA all consisted of approximately 500 g. All of the received test material was ground using an IKA M20 mill (Staufen, Germany), resulting in a maximum particle size of 0.5 mm. After each sample grinding, the mill was cleaned as follows: rests of flour were removed with a laboratory vacuum cleaner, and then the mill was rinsed with ultrapure water and acetone. The powdered sample material was introduced into large, clean plastic buckets and homogenized by manual shaking. For analysis, 10 g (+/- 0.1 g) of the homogenized test material were weighed using a precision balance (AT261 from Mettler-Toledo, Zurich, Switerland), introduced into a glass Erlenmeyer, and incubated in 40 ml of acetonitrile. Samples were mixed with an ultra-turrax mixer for 3 min and centrifuged for 10 min at 4000 g. 1 ml of the supernatant was diluted 1:4 with water, introduced into an injection vial, and submitted to UHPLC-MS/MS analysis.

### 2.4 Sample processing

In order to investigate the parameters that could influence epimerization (transformation from R-form into S-form), blank samples of wheat, triticale, rye and fodder pellets were spiked with 100 μg/kg (addition of 1 ml of a standard solution at 1 mg/l) of all alkaloids at 100 % R-form, and processed as follows:

#### 2.4.1 Influence of pH

10 g of sample were introduced into a glass Erlenmeyer, spiked as described before and covered with 10 ml of borate buffer (pH = 10), purified water (pH = 6.8) or sodium acetate buffer (pH = 3). After this, the samples were incubated in the dark at room temperature for 2 h before being extracted as described before and submitted to UHPLC-MS/MS analysis.

#### 2.4.2 Influence of heat

10 g of sample were introduced into a glass Erlenmeyer, spiked as described before and incubated at 100 °C for 1, 2 and 3 h. After this, the sample was left to cool down at room temperature, extracted as described before and submitted to UHPLC-MS/MS analysis.

#### 2.4.3 Influence of heat and humidity

10 g of sample were introduced into a glass Erlenmeyer, spiked as described before, covered with 10 ml of water and mixed by slight shaking. Then, the samples were incubated at 100 °C for 1, 2 and 3 h. After this, the sample was left to cool down at room temperature, extracted as described before and submitted to UHPLC-MS/MS analysis.

#### 2.4.4 Influence of UV light

10 g of sample were introduced into a glass Erlenmeyer and spiked as described before. Then, the samples were submitted to UV-light (302 nm) using a UV 2000 transilluminator from BioRad (Hercules, CA, USA) for 1, 2 and 3 h. After this, the sample was extracted as described before and submitted to UHPLC-MS/MS analysis.

All tests were carried out in triplicate.

### 2.5 UHPLC-MS/MS analysis

Ergot alkaloids were analysed using a Waters Acquityi-class UHPLC-system with a WatersXevo TQs mass spectrometer. The instrument was equipped with a Waters Acquity BEH C18 column (130 Å, 1.7 μm, 2.1 mm × 100 mm). Mobile phases were water (A) and acetonitrile (B) with 0.1 % (v/v) formic acid. The flow was set to 0.4 ml/min and the gradient was as follows: 0–3 min: isocratic conditions at 90% A; from 3 to 6 min: to 70 % A; isocratic conditions of 70 % A kept until 9 min; from 9 to 11 min: to 50 % A; from 11 to 14 min: to 10 % A; from 14 to 15 min: back to initial conditions. The detection of the alkaloids was done using following transitions: ergocristine/ergocristinine: 592.4 > 223.3 and 592.4 > 305.4; ergotamine/ergotaminine: 582.5 > 208.2 and 564.4 > 223.2; ergokryptine/ergokryptinine: 558.5 > 305.3 and 558.5 > 223.3; ergocornine/ergocorninine: 562.5 > 223.3 and 544.4 > 277.5; ergosine/ergosinine: 548.3 > 223.2 and 548.3 > 208.1; ergométrine/ergométrinine: 326.3 > 223.3 and 326.3 > 208.2. The first transition of each compound was used as quantifier. The source was heated at 150 °C, desolvation flow (N_2_) was set to 800 l/h at 450 °C and cone flow was set to 150 l/h 5 μl of diluted sample extract were injected. Using these conditions, all alkaloids and epimers were separated (Figure 1A and B).

### 2.6 Validation

The method validation was done on flours obtained from different cereals, namely wheat, triticale and rye. No ergot alkaloid at concentrations above the limit of detection was detected in any sample.

Linearity was tested by spiking blank samples (samples with amounts of ergot alkaloids below the limit of detection) of each flour with different amounts of ergot alkaloids (0, 1, 5, 50, 100, 250 and 500 μg/kg) and analyzed as described above. In order to assess the specificity of the method, the absence of parasite peaks on the chromatograms was verified. The matrix-effect was investigated by comparing the slopes of the matrix-matched calibration curves with the slope of non-matrix-matched calibration curves.

RSD_r_, RSD_R_, accuracy and recovery were tested by spiking blank samples of wheat flour with 5 and 100 μg/kg of each alkaloid, extracted and analyzed as described above. Recovery was verified by calculating the amount of measured alkaloid using the calibration equation and comparing this value to the amount of spiking. For the determination of accuracy, the measured alkaloid concentration was corrected for recovery and then compared with the amount of spiking.

The limit of detection (LD) was defined according to the guidelines of the European Reference Laboratory for mycotoxins and plant toxins: a blank sample was extracted and analyzed 10 times. The LD was than calculated using the formula LD = 3.9 x σ/b with σ being the standard deviation of the blank signals and b the slope of the calibration curve. The LQ consists of 3.3 times the LD.

### 2.7 Statistics

An ANOVA test (in the case of a normal distribution of the values) or a Kruskal–Wallis test (in the opposite case) was done on the calculated average concentrations. Normality of the distribution was tested with a Shapiro–Wilk test. The resulting P values (two-way ANOVA test) show if the risk that the observed variations are due to random sampling is less than 5 % (when the P value is below 0.05).

## 3 Results and discussion

### 3.1 Validation

All validation data are given in Table 1 and are discussed below. Values reported for repeatability, reproducibility, accuracy and recovery are means.

**Table 1:** Performance characteristics of the analytical method.

	Matrix effect					
	Slope without matrix	Linearity (r^2^)	Slope in wheat	Slope in triticale	Slope in rye	CV of slopes
ergosine	2205.3	0.9959	2250.4	2313.5	2299.1	2.17%
ergosinine	1998.4	0.9945	1987.4	2015.4	1784.0	5.59%
ergocornine	1196.9	0.9963	1223.4	1269.1	1211.5	2.55%
ergocorninie	1248.7	0.9931	1347.2	1124.9	1245.7	7.33%
α-ergocryptine	571.1	0.9974	598.9	628.0	635.7	4.85%
α-ergocryptinine	487.8	0.9998	451.1	422.0	475.4	6.33%
ergotamine	954.0	0.9969	1019.9	1044.5	1019.2	3.84%
ergotaminine	1010.1	0.9947	1115.1	1242.1	1098.7	8.56%
ergometrine	3397.9	0.9967	3033.4	2993.1	2772.0	8.51%
ergometrinine	2658.4	0.9925	2547.9	2731.4	2457.8	4.64%
ergocristine	231.1	0.9982	238.8	244.2	245.2	2.7%
ergocristinine	258.4	0.9974	298.5	264.7	269.9	6.49%

	RSD_r_ 5 μg/kg	RSD_r_ 100 μg/kg	RSD_R_ 5 μg/kg	RSD_R_ 100 μg/kg	recovery 5 μg/kg	recovery 100 μg/kg
ergosine	11.35%	16.21%	17.21%	15.87%	78.45%	92.34%
ergosinine	12.45%	16.36%	18.55%	16.54%	86.87%	90.14%
ergocornine	11.11%	17.16%	19.55%	17.7%	78.62%	101.4%
ergocorninie	9.45%	15.03%	16.54%	19.47%	77.56%	89.64%
α-ergocryptine	15.93%	17.13%	17.21%	18.95%	92.06%	93.47%
α-ergocryptinine	10.08%	18.46%	15.47%	17.54%	80.06%	94.7%
ergotamine	14.46%	17.17%	12.94%	14.48%	80.36%	87.45%
ergotaminine	8.69%	16.48%	11.45%	13.25%	93.24%	86.96%
ergometrine	8.03%	12.86%	17.39%	16.58%	83.07%	96.47%
ergometrinine	13.34%	15.42%	13.67%	16.47%	86.54%	96.08%
ergocristine	14.89%	14.77%	11.01%	18.04%	89.13%	94.69%
ergocristinine	11.68%	11.89%	14.48%	11.58%	76.54%	100.06%

	Accuracy 5 μg/kg	Accuracy 100 μg/kg	LD (μg/kg)	LQ (μg/kg)
ergosine	101.45%	103.45%	0.42	1.42
ergosinine	96.54%	108.57%	0.64	2.65
ergocornine	96.03%	92.45%	0.43	1.14
ergocorninie	97.93%	94.65%	0.34	3.01
α-ergocryptine	90.21%	93.84%	0.35	1.71
α-ergocryptinine	101.63%	99.41%	0.60	1.64
ergotamine	105.00%	105.46%	0.52	1.25
ergotaminine	109.68%	106.32%	0.47	1.04
ergometrine	93.87%	97.34%	0.66	2.16
ergometrinine	97.98%	96.8%	0.93	2.47
ergocristine	107.64%	93.71%	0.38	1.38
ergocristinine	99.72%	101.2%	0.78	3.54

**Figure 2:**
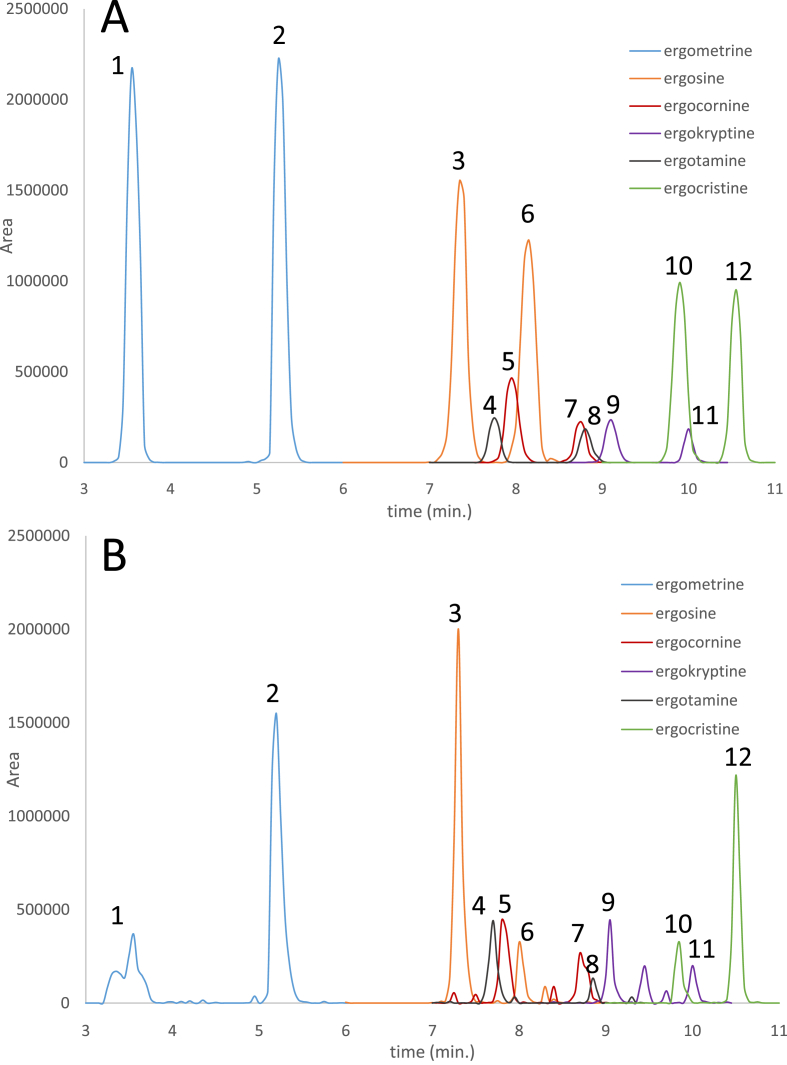
Chromatograms of a standard solution at 25 μg/L (A) and an extract of a processed cereal sample (1 h at 100 °C) spiked at 100 μg/kg (B): 1 = ergometrine; 2 = ergometrinine; 3 = ergosine; 4 = ergotamine; 5 = ergocornine; 6 = ergosinine; 7 = ergocorninine; 8 = ergotaminine; 9 = ergokryptine; 10 = ergocristine; 11 = ergokryptinine; 12 = ergocristinine.

Calibration was done from 1 to 500 μg/kg, and coefficients of correlation were all above 0.99 in every matrix, so the linearity was confirmed. The chromatograms of the blank samples didn't show any peak for the respective retention times, indicating that the method is very specific and the risk of false positives is very low. The slopes of the calibration curves in spiked blank wheat and in pure solvent (acetonitrile) diverge by less than 10 %, showing that there is no significant matrix effect for any compound. Therefore, it was decided that quantification could be done using non matrix-matched external calibration. Separation of all ergot alkaloids and the corresponding epimers was sufficient for allowing separate integration of the peaks (see Figure 2, showing a chromatogram of a standard solution in acetonitrile (figure 2 A) and a blank wheat extract spiked at 100 μg/kg (Figure 2 B) on which the peaks of each alkaloid and each corresponding epimer are clearly distinguishable).

RSD_r_ and RSD_R_ are all below 20 % and accuracy and recovery values are all higher than 70 %. LODs range from 0.34 μg/kg to 0.93 μg/kg and LOQs range from 1.04 μg/kg to 3.54 μg/kg. For simplicity reasons, default values of 1 μg/kg (LOD) and 5 μg/kg (LOQ) are used for all compounds in this study.

All validation data of this study are comparable to those of most previously published methods (Schummer et al., 2018), and the method is suitable for the intended analyses of ergot alkaloids in cereal-based feed. This also shows that dilution instead of lengthy clean-up steps is sufficient to allowing precise quantification of all ergot alkaloids and corresponding epimers without matrix effect, while at the same time it allows working much faster and cheaper as no SPE or other purification consumables are needed.

### 3.2 Epimerization

#### 3.2.1 Influence of pH

The epimerization rates after incubation at different pH values are given in Table 2A.

**Table 2:** Percentage of S-form measured after different processings of spiked samples.

			ergosinine	ergocorninie	ergocryptinine	ergotaminine	ergometrinine	ergocristinine
A - incubation at different pH	wheat	pH = 7	0%	29%	4%	0%	10%	50%
		pH = 3	0%	30%	5%	0%	17%	49%
		pH = 10	0%	45%	10%	0%	21%	53%
	triticale	pH = 7	0%	27%	6%	0%	0%	43%
		pH = 3	0%	26%	0%	0%	11%	44%
		pH = 10	0%	43%	0%	0%	17%	56%
	rye	pH = 7	0%	36%	7%	0%	15%	66%
		pH = 3	0%	37%	9%	0%	19%	68%
		pH = 10	5%	49%	15%	5%	25%	66%
	fodder pellets	pH = 7	0%	38%	8%	0%	22%	67%
		pH = 3	0%	38%	8%	0%	24%	66%
		pH = 10	5%	50%	12%	0%	33%	70%
B - heating for 1, 2 and 3 h	wheat	1 h	1%	78%	29%	0%	54%	91%
		2 h	2%	83%	40%	0%	78%	92%
		3 h	3%	84%	44%	3%	77%	94%
	triticale	1 h	10%	84%	44%	3%	59%	92%
		2 h	11%	82%	48%	6%	69%	94%
		3 h	11%	87%	51%	8%	68%	95%
	rye	1 h	11%	77%	40%	8%	65%	92%
		2 h	12%	81%	49%	4%	72%	94%
		3 h	12%	83%	47%	7%	75%	95%
	fodder pellets	1 h	9%	81%	42%	8%	69%	92%
		2 h	10%	85%	43%	9%	68%	95%
		3 h	11%	86%	45%	10%	72%	96%
C - heating in the presence of water	wheat	1 h	0%	73%	26%	1%	46%	85%
		2 h	0%	75%	27%	0%	71%	90%
		3 h	0%	75%	31%	1%	78%	91%
	triticale	1 h	1%	75%	31%	0%	58%	92%
		2 h	1%	82%	45%	0%	69%	93%
		3 h	0%	83%	45%	0%	64%	94%
	rye	1 h	1%	73%	35%	0%	51%	91%
		2 h	0%	75%	36%	0%	70%	90%
		3 h	0%	76%	37%	0%	73%	92%
	fodder pellets	1 h	0%	73%	26%	0%	31%	88%
		2 h	1%	74%	26%	0%	53%	91%
		3 h	1%	77%	34%	0%	54%	93%
D - illuminating with UV light	wheat	1 h	0%	40%	11%	0%	2%	74%
		2 h	0%	53%	15%	0%	6%	82%
		3 h	0%	56%	18%	0%	7%	83%
	triticale	1 h	0%	54%	25%	0%	6%	80%
		2 h	0%	56%	19%	0%	17%	80%
		3 h	0%	64%	24%	0%	13%	88%
	rye	1 h	0%	39%	9%	0%	8%	74%
		2 h	0%	57%	18%	0%	12%	83%
		3 h	0%	59%	18%	0%	14%	84%
	fodder pellets	1 h	0%	67%	18%	0%	4%	90%
		2 h	0%	70%	24%	0%	10%	90%
		3 h	0%	68%	22%	0%	13%	87%

After incubation of spiked samples in solutions at different pH values, it was observed that ergotamine and ergosine stayed in the R-form for nearly 100 % - only at pH = 10, 5 % of the detected alkaloids were in S-form for rye and fodder pellets. Ergometrine also stayed mostly in the R-form as the part of S-form ranged between 0 and 15 % while ergocristine mostly passed into S-form with rates between 43 and 70 %. This suggests that epimerization doesn't happen to the same extend for all alkaloids. For alkaloids undergoing epimerization, the highest epimerization rates were always observed for pH 10, however this observation is not statistically significant at p < 0.05 (p = 0.1698) and could be due to chance. Also, no statistically significant differences between incubations in alkaline or acid buffers and water were observed (p = 0.4394 and 0.1716). In this context it is important to consider the pK_a_-values of the alkaloids, that range from 5.5 (ergocristine) to 6.0 (ergometrine) for the –*ines* and from 4.8 (ergocorninine) to 6.2 (ergometrinine) for the –*inines*, as well as the acidity of the proton next to the carboxamide group. The presence of the proton is important because electronegative substituents near the carboxamide group act to increase the acidity, and might influence the epimerization. Indeed, ergot alkaloids thus are positively charged at N-6 at low pH values and neutral at basic pH values and it may be that alkaloids with lower pK_a_ values like ergocristine are favourable to epimerization and alkaloids with higher pK_a_ values like ergometrine are less favourable to it. This would be in line with the observations of the present study. Another factor that must be considered is steric hindrance. In fact, in contact with protic solvents at different pH values, a hydrogen atom is added or removed from the molecule with resultant changes in structure. This change might cause “folding” of the molecule such that into the intermediate state between the S and the R-configuration (see Figure 1) is mechanically hindered for the preferred configuration, as observed before by Andrae et al. (2014). Thus, steric hindrance may prevent some alkaloids undergoing epimerization even though their pKa value would favour it.

#### 3.2.2 Influence of heat

The epimerization rates after incubation at 100 °C for different time periods are given in Table 2B.

The table shows that ergotamine and ergosine are the most stable alkaloids, though this time the epimerization rate is slightly higher with up to 10 % (ergotamine) and 12 % (ergosine). Ergometrine and ergocryptine, that showed high epimeric stability after incubation in water and buffers, are considerably less stable when heated, as up to 51 % (ergocryptine) and 78 % (ergometrine) pass into the corresponding S-form. Concerning ergocornine and ergocristine that already showed quite low epimeric stability in protic solvents, the percentage of transformation into the S-form increased with rates between 77 % and 87 % (ergocornine) resp. 91 % and 96 % (ergocristine).

The number of repetitions and thus the number of observations may be too low to show statistically significant behaviour, but generally, the present results suggest that epimerization is favoured by heating. Furthermore, this is in line with previous studies that showed that baking has a positive effect on the transformation of the R-form epimers to the S-form (Mainka et al., 2005; Lampen and Klaffke, 2006; Lauber et al., 2005; Hafner et al., 2008; EFSA, 2012; Merkel et al., 2012). Again, the epimerization rates of the ergot alkaloids after exposure to heat vary considerably in-between the alkaloids. Also, it seems that epimerization occurs quite fast after exposure to heat, as no difference between heating for 1, 2 or 3 h was observed. Concerning inter-matrix variability, no significant differences were observed.

Heating seems to have a much stronger effect on the epimerization than protic solvents as all alkaloids undergo epimerization even though the percentage may be low, and for ergocornine and above all ergocristine, most of the alkaloids passed into the less toxic S-form after exposure to heat.

#### 3.2.3 Influence of heat and humidity

The epimerization rates after incubation in water at 100 °C for 1, 2 and 3 h are given in Table 2C.

No differences were observed for ergocornine, ergocryptine, ergometrine and ergocristine compared to the action of heat alone (p > 0.05). Thus, for ergosine and ergotamine, the alkaloids showing the highest epimeric stability in the previous tests, no epimerization was observed any more, contrarily to the action of heat alone where epimerization rates of about 10 % were observed. This observation is not due to chance, as the differences between both tests are statistically significant (p-values of <0.00001). Therefore, for epimerization, dry heating should be preferred to heating in the presence of water. A possible explanationfor the inhibition of epimerization in the presence of water may be that water acts on the matrix and thus induces the formation of bindings of the alkaloids with matrix components that hamper epimerization. It can be excluded that the presence of water retards the heating of the sample, as even after 1 h all water is evaporated and, as the previous tests show no difference between heating for 1, 2 or 3 h, the remaining time would be enough to allow epimerization.

#### 3.2.4 Influence of UV light

The epimerization rates after irradiation by UV light (302 nm) for 1, 2 and 3 h are given in Table 2D.

Also after incubation in UV light, the R-forms of ergotamine and ergosine are very stable, no epimerization could be observed. By contrast, the epimerization of ergocristine is almost complete as epimerization rates of 74 %–90 % are observed. Ergometrine and ergocryptine show slight epimerization rates of 2 %–17 % resp. 9 %–25 %, while ergocornine shows medium rates of 39 %–70 %. Again, no differences in-between the matrices were observed. The highest epimerization rates were measured after 2 h of exposure to light, however these variations are not statistically significant (p = 0.3407 and 0.4548).

#### 3.2.5 Synthesis of the epimerization tests

The previous results suggest that the configuration of the asymmetry centre on the C_8_-atom of the studied alkaloids can be changed under certain circumstances, like water, UV-light or temperatures of 100 °C, and thus the epimeric configuration of the alkaloids can be modified. The strongest influence on the epimerization is heating at 100 °C for at least 1 h, and for ergocristine, ergocornine, ergocryptine and ergometrine, the epimerization rates are strong or very strong. However if water is added to the samples prior to heating, the epimerization rates decrease slightly.

Ergotamine and ergosine are very stable in their respective R-form and show no distinctive tendency to epimerization.

The observations made on spiked, blank samples thus give quite interesting observations. However, the observations must be confirmed on naturally contaminated samples as it may be that the behaviour is not as pronounced because alkaloids may not be as accessible (partially hidden in matrix components; less accessible for UV-light, water and heat, etc) as in spiked samples.

As noted above, some explanation of possible epimerization mechanisms at different pH values can be proposed. However it is much more difficult to deduce the mechanism(s) that favour epimerization in the presence of heat or UV-light, and further studies are now required to investigate these mechanisms.

### 3.3 Epimerization of naturally contaminated feed samples

In order to verify if the conclusions drawn above remain true in naturally contaminated samples, seven cereal-based feed samples (A – G; 3 samples of triticale, 2 samples of wheat, 1 sample of rye and 1 sample of fodder pellets) were analyzed with the previously described accredited method and total concentrations of all alkaloids of 47 μg/kg to 822 μg/kg were measured (Table 3). All alkaloids were detected at least 4 times, and the maximum concentrations of the individual alkaloids (sum of R- and S-epimers) range from 92 for ergometrine/inine to 444 μg/kg for ergocristine/inine, both in wheat. These concentrations are in line with previous studies on different cereals (Ruhland and Tischler, 2008; Di Mavungu et al., 2012; Arroyo-Manzanares et al., 2018; Schummer et al., 2018). The highest concentrations were found in wheat samples and not in rye or triticale, which are, according to literature (e.g. Krska et al., 2008; Di Mavungu et al., 2012), more predestinated for ergot contamination due to their open florets. However, the number of samples is too low to perform consistent statistical evaluations of these findings. Nonetheless it confirms previous findings that not only rye but also wheat and other cereals should be included in monitoring programs and risk assessments (Schummer et al., 2018).

**Table 3:** Concentrations of ergot alkaloids (sum of epimers) of unprocessed and processed feed.

	Sample		ergocristine/inine	ergotamine/inine	ergokryptine/inine	ergocornine/inine	ergosine/inine	ergometrine/inine	Total alkaloid concentration
triticale	A	no processing	48	24	<1	<1	5	<5	77
		100 °C	<5	<5	8	<5	5	<5	13
		pH = 10	<5	<5	6	13	23	9	51
		UV-light	24	<5	<1	5	23	<5	52
	B	no processing	139	73	50	109	124	14	509
		100 °C	107	36	25	98	112	<5	378
		pH = 10	137	24	38	105	137	<5	441
		UV-light	290	53	67	122	176	<5	708
	C	no processing	<1	<1	15	32	<5	<1	47
		100 °C	<1	<1	7	29	10	<5	46
		pH = 10	<1	<1	<5	6	<5	<5	6
		UV-light	<1	<1	13	32	7	<5	52
wheat	D	no processing	14	7	269	66	88	64	508
		100 °C	30	<5	125	119	141	7	422
		pH = 10	12	8	53	24	61	7	165
		UV-light	140	44	73	22	67	<5	346
	E	no processing	444	130	45	86	25	92	822
		100 °C	186	80	14	43	58	26	407
		pH = 10	248	35	15	45	125	37	505
		UV-light	509	46	8	22	49	11	645
rye	F	no processing	<5	182	6	16	25	16	245
		100 °C	<5	288	<5	8	17	7	320
		pH = 10	<1	81	<5	26	46	8	161
		UV-light	6	224	<5	10	14	11	265
fodder pellets	G	no processing	9	<5	33	154	46	<1	242
		100 °C	<5	<1	<1	<1	<1	<1	<5
		pH = 10	<5	<1	14	<5	<5	<1	14
		UV-light	11	33	<5	24	119	33	220

After the first analysis and the determination of the initial concentrations of ergot alkaloids, the feed samples were processed as previously described (heating at 100 °C, incubation in borate buffer at pH = 10 and submission to UV-light for 1 h) and reanalyzed (Table 4).

**Table 4:** Percentages of the respective S-epimer of unprocessed and processed feed.

	Sample		ergocristine/inine	ergotamine/inine	ergokryptine/inine	ergocornine/inine	ergosine/inine	ergometrine/inine
triticale	A	no processing	32%	2%	0%	0%	2%	13%
		100 °C	52%	8%	8%	47%	3%	49%
		pH = 10	52%	5%	29%	29%	4%	26%
		UV-light	81%	4%	0%	7%	3%	14%
	B	no processing	42%	3%	38%	27%	4%	12%
		100 °C	75%	5%	20%	48%	5%	42%
		pH = 10	75%	4%	7%	32%	4%	12%
		UV-light	65%	3%	6%	20%	3%	17%
	C	no processing	0%	0%	53%	13%	6%	0%
		100 °C	0%	0%	22%	63%	4%	54%
		pH = 10	0%	0%	51%	27%	5%	0%
		UV-light	0%	0%	10%	21%	4%	0%
wheat	D	no processing	19%	2%	62%	19%	3%	8%
		100 °C	38%	8%	10%	57%	3%	50%
		pH = 10	39%	3%	3%	19%	4%	14%
		UV-light	31%	2%	4%	13%	4%	7%
	E	no processing	38%	2%	43%	29%	3%	22%
		100 °C	78%	3%	24%	72%	3%	49%
		pH = 10	39%	2%	4%	19%	3%	15%
		UV-light	62%	2%	16%	19%	4%	24%
rye	F	no processing	34%	3%	12%	11%	3%	20%
		100 °C	50%	3%	13%	17%	3%	32%
		pH = 10	0%	4%	12%	27%	4%	18%
		UV-light	33%	2%	10%	15%	4%	20%
fodder pellets	G	no processing	49%	2%	8%	7%	31%	0%
		100 °C	59%	0%	0%	0%	0%	0%
		pH = 10	2%	0%	2%	53%	7%	0%
		UV-light	80%	1%	10%	7%	3%	21%

Previous studies report that baking reduces the concentrations of ergot alkaloids in food (Mainka et al., 2005; Lampen and Klaffke, 2006; Lauber et al., 2005; Hafner et al., 2008; Merkel et al., 2012, 2013). Therefore, this hypothesis was evaluated, and actually the results of the present study show punctual decreases of concentrations, e.g. the decreases of the total alkaloid concentrations of 77 μg/kg to 13 μg/kg in sample A, of 822 μg/kg to 407 μg/kg in sample E and of 2402 μg/kg to <5 μg/kg in sample G. However, total concentrations of other samples remained more or less unchanged (samples C and D) or even increased (from 245 μg/kg to 320 μg/kg in sample F). As a result, no statistically significant decrease of total alkaloid concentrations could be observed (p = 0.1788), and it may be that the observed variations are due to random sampling. Also, since it is well known that inhomogeneity of the samples is a huge problem for ergot alkaloid analyses it cannot be ruled out that the variations are also partially due to inhomogeneous samples. Indeed, despite very careful milling and blending of the samples, some single black spots remained detectable in the sample powder. These could be small fragments of sclerotias that could have interfered with the results. Thus, as previously said, all tests were carried out in triplicate and all interpretations were done on the means of the measured concentrations. This corrects for inhomogeneity to a certain extent but it cannot be fully excluded that a small bias still results from inhomogeneity. It may be advised to repeat some of these tests in a study carried out at larger scale, with more samples, and maybe more repetitions (more than triplicate) in order to fully exclude the bias resulting from possible inhomogeneity of the samples.

Similar observations were made after incubation of the samples in alkaline buffer and UV light: punctual decreases were observed, above all for the incubation in alkaline buffer (e.g. decreases of 52 μg/kg to 6 μg/kg in sample C or of 508 μg/kg to 165 μg/kg in sample D), but no statistically significant decreases were observed (p-values of 0.1233 and 0.4376). This is in opposition to the observations of Rehácek and Sajdl (1990; in Komarova and Tolkachev, 2001) who observed that ergot alkaloids are photosensitive and can produce lumi derivatives through adsorption of water to the C_9_–C_10_ double binding and thus reduce the concentrations of free ergot alkaloids. It might also be possible that the extraction procedure used in this study is effective enough to break these bindings so that all alkaloids are free in the extract.

It was also investigated if the findings obtained by analyzing spiked samples on the modification of the R/S ratio of the ergot alkaloids remain true in naturally contaminated samples (Table 4).

The first observation is that ergotamine and ergosine are very stable in their R-form, nearly no S-form was detected and thus no significant epimerization for these two compounds could be observed for no processing, so neither for heating, incubation in alkaline buffer nor by illuminating the samples. This confirms the findings made above on spiked samples.

**Figure 3:**
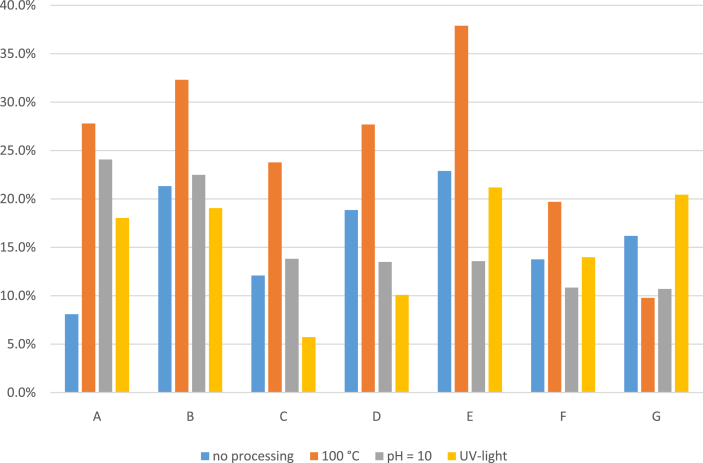
Average percentages of S-epimers of all alkaloids of unprocessed and processed feed.

For ergocristine, ergocornine and ergometrine, it can also be confirmed that heating has an influence on the epimerization as the percentage of S-form increases significantly after heating of the sample at 100 °C for one hour (p = 0.0060 for ergocristine; p = 0.0101 for ergocornine; p = 0.00001 for ergometrine. Samples with concentrations < LOD were ignored). Interestingly, this could not be confirmed for ergokryptine, as in this case the R/S ratio increased after heating in 5 out of 7 cases (samples B, C, D, E, G). This is in opposition to the observations of Merkel (2013) who observed that heating decreases the R/S ratio of all alkaloids. However, the increase of the R/S ratio for ergokryptine might be due to back-epimerization from ergokryptinine (as it was present in the naturally contaminated sample). The present data do not allow explaining why the epimerization of ergokryptine in naturally contaminated samples is different than in spiked samples, though it may be linked to matrix effects or bindings of the molecules to matrix components that cause steric hindrance hampering the epimerization. Nonetheless, when comparing the average R/S ratios before and after heating for 1 h in all samples (Figure 3), it can be confirmed that the global ratio decreases significantly (p = 0.0177).

Statistically significant decreases of the R/S ratios could also be observed for ergocornine after incubation in alkaline buffer (p = 0.0167) and for ergocristine and ergokryptine after illumination (p = 0.0210 and 0.0071 respectively). No statistically significant tendencies were observed for ergometrine and for the average R/S ratio of all alkaloids. In fact this may be due to the fact that the test population was too small. Nonetheless, the observations that heating, incubation in alkaline buffer and UV light influence the R/S ratio of four out of six of the major ergot alkaloids, have been partially confirmed on naturally contaminated samples, but these are not strong enough to significantly change the R/S configuration of ergot alkaloids and thus to turn toxic into nontoxic feed. It was tried to investigate if a relationship between the stability of the alkaloid and its respective structure exists, however no correlation could be identified. This may be due to the low amount of data but might be interesting to investigate in a follow-up study.

Generally, the processing tests carried out in naturally contaminated samples confirm the findings carried out on blank, spiked samples: water, UV-light and temperatures of 100 °C favour epimerization, at least for ergocristine, ergocornine, ergocryptine and ergometrine. Ergotamine and ergosine are very stable in their respective R-form and epimerization rates are low. This confirmation is very important as the ergot fragments in these samples are less available (partially protected from light, heat and water) than those in spiked samples, and it shows that testing on blank, spiked samples where inhomogeneity is not a problem, gives accurate results.

## 4 Conclusions

The present study suggests that ergotamine and ergosine are very stable ergot alkaloids, and neither their concentrations, nor their respective R/S ratios, are significantly influenced by heating, protic solvents or UV light. In contrast, ergocristine, ergokryptine, ergocornine and ergometrineare influenced by these parameters: heating can decrease the concentrations of these alkaloids and heat, protic solvents and UV light influence the R/S ratio towards the S-form, though the respective influence on the epimerization of these compounds is variable. A follow-up study conducted at larger scale will be necessary to further investigate these findings and to strengthen the conclusions.

The findings of this study also suggest that, although reducing the total concentration of all ergot alkaloids and influencing the epimerization towards the less toxic S-form are possible, these effects are not strong enough to change the composition of ergot alkaloids in feed substantially and to transform toxic feed into non-toxic feed. Nonetheless, during laboratory analyses, protic solvents should be avoided and the sample should not be heated or submitted to light for long periods in order to give accurate results for the respective R- and S-epimers and thus allowing coherent risk assessment.

## Declarations

### Author contribution statement

Claude Schummer: Conceived and designed the experiments; Analyzed and interpreted the data; Wrote the paper.

Irène Zandonella: Performed the experiments; Analyzed and interpreted the data.

An van Nieuwenhuyse: Contributed reagents, materials, analysis tools or data.

Gilbert Moris: Conceived and designed the experiments; Contributed reagents, materials, analysis tools or data.

### Funding statement

This research did not receive any specific grant from funding agencies in the public, commercial, or not-for-profit sectors.

### Competing interest statement

The authors declare no conflict of interest.

### Additional information

No additional information is available for this paper.
